# Genome sequences of *Vibrio harveyi* VH2 and *Vibrio harveyi* Vhp1-sp

**DOI:** 10.1128/mra.00087-25

**Published:** 2025-06-09

**Authors:** Stavros Droubogiannis, Andriana Triga, Michail Pavlidis, Pantelis Katharios

**Affiliations:** 1 Department of Biology, School of Sciences and Engineering, University of Crete, Heraklion, Greece; 2 Institute of Marine Biology, Biotechnology & Aquaculture, Hellenic Centre for Marine Research, Heraklion, Greece; Montana State University, Bozeman, Montana, USA

**Keywords:** *V. harveyi*, genomic analysis, pathogens, aquaculture

## Abstract

*Vibrio harveyi* is a major bacterial pathogen of marine aquatic animals causing significant economic losses in aquaculture. Here, we present the complete genomes of two pathogenic strains, VH2 (5.84 Mb, 44.99% GC) and Vhp1-sp (6.30 Mb, 44.98% GC), which encode numerous virulence factors, resistance genes, and genomic islands.

## ANNOUNCEMENT

*Vibrio harveyi* is a serious opportunistic pathogen infecting a wide range of aquatic organisms (1, 2). Outbreaks of *V. harveyi* can cause substantial economic losses; however, the precise mechanisms underlying its pathogenicity remain unclear (2, 3). Here, we report the complete genomes of two pathogenic *V. harveyi* strains, VH2 and Vhp1-sp, which were isolated from *Seriola dumerili* and *Dicentrarchus labrax,* respectively, in Greece.

*V. harveyi* strains VH2 and Vhp1-sp were isolated from the kidneys of moribund fish during vibriosis outbreaks in aquaculture facilities in Greece. Samples were streaked onto Lysogeny Broth (10 g L^−1^ tryptone, 5 g L^−1^ yeast extract, 10 g L^−1^ NaCl, 1 L deionized water, 0.75 g L^−1^ MgSO_4_, 1.5 g L^−1^ KCl, and 0.73 g L^−1^ CaCl_2_) (LB) and Thiosulfate-Citrate-Bile-Sucrose (TCBS) agar, with subculturing to ensure purity. Bacterial strains were stored in MicroBank tubes (Pro-Lab Diagnostics, Richmond Hill, Canada) at −80°C. The same growth conditions were used for bacterial cultures intended for genomic DNA extraction.

Genomic DNA was extracted using the DNeasy Blood and Tissue Kit (Qiagen, Hilden, Germany) according to the manufacturer’s instructions. DNA quality and concentration were assessed using a NanoDrop spectrophotometer (Thermo Fisher Scientific, Waltham, MA, USA) and Qubit fluorometer (Thermo Fisher Scientific, Waltham, MA, USA). Short-read libraries were prepared using the BGI Optimal Library Prep Kit and sequenced using the DNBseq platform (BGI Tech Solutions, Hong Kong), with 150 bp paired-end reads. Library preparation included DNA fragmentation, end-repair, adapter ligation, size selection, and PCR amplification. Read quality control was conducted using SOAPnuke 2X (4) to remove low-quality reads and adapter sequences. Long-read libraries were prepared using the Oxford Nanopore Technologies’ Ligation Sequencing Kit (SQK-LSK110). Genomic DNA (~1 µg) was size-selected using magnetic beads to enrich for high-molecular-weight fragments. Library preparation included end-repair, dA-tailing, ligation of barcode adapters, magnetic bead purification, and adapter ligation. Sequencing was performed on the PromethION platform using R9.4.1 flow cells. Genomic DNA was not enzymatically or mechanically sheared prior to library preparation to preserve long read lengths. Base calling was carried out using Guppy v5.0. Reads with mean quality <9 or length <2,000 bp were filtered using Porechop v0.2.4 with default settings.

Hybrid *de novo* assemblies were generated using Flye v2.9 for VH2 and Unicycler v0.4.8 for Vhp1-sp via the BV-BRC web platform (5, 6). The quality of assembly was assessed using Quast v5.0.2 (7). Circularization was handled automatically by the assemblers. Annotation was performed using the RAST tool kit (RASTtk) (5) and the NCBI Prokaryotic Genome Annotation Pipeline (PGAP) (8). Genomic islands were predicted using IslandPick, SIGI-HMM, and IslandPath-DIMOB through the IslandViewer4 webserver (9). Species confirmation was done using average nucleotide identity (ANI) analysis against *V. harveyi* references. Long- and short-read sequencing statistics along with the assembly statistics for both strains are summarized in Table 1. Default parameters were used unless stated otherwise.

**TABLE 1:** Genome description of *V. harveyi* VH2 and *V. harveyi* Vhp1-sp

Feature	*V. harveyi* VH2	*V. harveyi* Vhp1-sp
Total genome size (bp)	5,836,028	6,295,716
GC content (%)	44.99	44.98
Number of chromosomes	2	2
Number of plasmids	None	2
Plasmid sizes (bp)	–^*a*^	76,688; 76,179
Protein-coding sequences	5,300	5,883
tRNA genes	133	133
rRNA genes	37	34
Hypothetical proteins	1,257	1,527
Genomic islands	72 (37 Chr1, 35 Chr2)	64 (42 Chr1, 22 Chr2)
Long-read sequencing		
Reads number #	186,898	402,656
Reads total bases (bp)	2,763,918,301	3,000,003,347
Reads mean length (bp)	14,788	7,450
Reads N50 (bp)	21,716	10,294
Reads N90 (bp)	7,285	3,412
Reads max length (bp)	234,683	64,768
Reads min length (bp)	2,000	2,000
Short-read sequencing		
Clean reads #	12,420,822	12,425,500
Clean reads total bases (bp)	1,242,082,200	1,242,550,000
Reads length (bp)	100;100	100;100
Q20 (%)	97.41	97.62
GC (%)	44.99	44.84
Assembly statistics		
Contigs #	2	4
Contig N50	3,534,894	3,752,363
Genome coverage	159.353	2.02

^*a*^ "–" indicates that the specific strain does not have any plasmid; therefore, plasmid size cannot be included.

The VH2 genome comprises two circular chromosomes totaling 5.8 Mb, with no plasmids encoding 5,300 coding sequences, 133 tRNAs, and 37 rRNAs. The Vhp1-sp genome spans 6.1 Mb with two chromosomes and two plasmids (76.7 kb and 76.1 kb), comprising 5,883 coding sequences, 133 tRNA genes, and 34 rRNA genes. Both genomes contain numerous hypothetical proteins and over 60 genomic islands linked to horizontal gene transfer. Virulence genes (e.g., hemolysins, metalloproteases, and T3SS), antibiotic resistance genes (*katG, qnrB*), and multiple defense systems (RM systems, GAPS, Zorya) were identified, emphasizing their adaptability and relevance to pathogenicity in aquaculture environments.

## Data Availability

The complete genome sequence of *Vibrio harveyi* VH2 is available in GenBank under accession numbers CP169261 and CP169262 for chromosomes 1 and 2, respectively. The associated BioProject and BioSample accession numbers are PRJNA290379 and SAMN03890867. The genome of *V. harveyi* Vhp1-sp is available under GenBank accession number JAIWIW000000000.

## AFTER EPUB

*[This article was published on 9 June 2025 with an error in the last author's affiliation. The affiliation was corrected in the current version, posted on 13 June 2025.]*

## References

[1] Triga A, Smyrli M, Katharios P. 2023. Pathogenic and opportunistic Vibrio spp. associated with vibriosis incidences in the Greek aquaculture: the role of Vibrio harveyi as the principal cause of vibriosis. Microorganisms 11:1197. doi:10.3390/microorganisms11051197 37317171 PMC10220884

[2] Zhang XH, He X, Austin B. 2020. Vibrio harveyi: a serious pathogen of fish and invertebrates in mariculture. Mar Life Sci Technol 2:231–245. doi:10.1007/s42995-020-00037-z 32419972 PMC7223180

[3] Atujona D, Cai S, Amenyogbe E. 2018. Mini review on Vibrio infection-a case study on Vibrio harveyi clade. Fish Aquac J 09:9–12. doi:10.4172/2150-3508.1000258

[4] Chen Y, Chen Y, Shi C, Huang Z, Zhang Y, Li S, Li Y, Ye J, Yu C, Li Z, Zhang X, Wang J, Yang H, Fang L, Chen Q. 2018. SOAPnuke: a MapReduce acceleration-supported software for integrated quality control and preprocessing of high-throughput sequencing data. Gigascience 7:1–6. doi:10.1093/gigascience/gix120 PMC5788068 29220494

[5] Davis JJ, Wattam AR, Aziz RK, Brettin T, Butler R, Butler RM, Chlenski P, Conrad N, Dickerman A, Dietrich EM, et al.. 2020. The PATRIC bioinformatics resource center: expanding data and analysis capabilities. Nucleic Acids Res 48:D606–D612. doi:10.1093/nar/gkz943 31667520 PMC7145515

[6] Kolmogorov M, Yuan J, Lin Y, Pevzner PA. 2019. Assembly of long, error-prone reads using repeat graphs. Nat Biotechnol 37:540–546. doi:10.1038/s41587-019-0072-8 30936562

[7] Gurevich A, Saveliev V, Vyahhi N, Tesler G. 2013. QUAST: quality assessment tool for genome assemblies. Bioinformatics 29:1072–1075. doi:10.1093/bioinformatics/btt086 23422339 PMC3624806

[8] Li W, O’Neill KR, Haft DH, DiCuccio M, Chetvernin V, Badretdin A, Coulouris G, Chitsaz F, Derbyshire MK, Durkin AS, Gonzales NR, Gwadz M, Lanczycki CJ, Song JS, Thanki N, Wang J, Yamashita RA, Yang M, Zheng C, Marchler-Bauer A, Thibaud-Nissen F. 2021. RefSeq: expanding the prokaryotic genome annotation pipeline reach with protein family model curation. Nucleic Acids Res 49:D1020–D1028. doi:10.1093/nar/gkaa1105 33270901 PMC7779008

[9] Bertelli C, Laird MR, Williams KP, Lau BY, Hoad G, Winsor GL, Brinkman FSL, Simon Fraser University Research Computing Group. 2017. IslandViewer 4: expanded prediction of genomic islands for larger-scale datasets. Nucleic Acids Res 45:W30–W35. doi:10.1093/nar/gkx343 28472413 PMC5570257

